# Gill function in an early arthropod and the widespread adoption of the countercurrent exchange mechanism

**DOI:** 10.1098/rsos.230341

**Published:** 2023-08-16

**Authors:** Jin-Bo Hou, Nigel C. Hughes, Melanie J. Hopkins, Degan Shu

**Affiliations:** ^1^ State Key Laboratory for Mineral Deposits Research, School of Earth Sciences and Engineering and Frontiers Science Center for Critical Earth Material Cycling, Nanjing University, Nanjing 210023, People's Republic of China; ^2^ Department of Earth and Planetary Sciences, University of California, Riverside, CA 92521, USA; ^3^ Division of Paleontology (Invertebrates), American Museum of Natural History, New York, NY 10024, USA; ^4^ Early Life Institute and State Key Laboratory of Continental Dynamics, Northwest University, Xi'an 710069, People's Republic of China

**Keywords:** Gill, respiration, computational fluid dynamics, metazoans, efficient mechanisms

## Abstract

Rising but fluctuating oxygen levels in the Early Palaeozoic provide an environmental context for the radiation of early metazoans, but little is known about how mechanistically early animals satisfied their oxygen requirements. Here we propose that the countercurrent gaseous exchange, a highly efficient respiratory mechanism, was effective in the gills of the Late Ordovician trilobite *Triarthrus eatoni*. In order to test this, we use computational fluid dynamics to simulate water flow around its gills and show that water velocity decreased distinctly in front of and between the swollen ends, which first encountered the oxygen-charged water, and slowed continuously at the mid-central region, forming a buffer zone with a slight increase of the water volume. In *T. eatoni* respiratory surface area was maximized by extending filament height and gill shaft length. In comparison with the oxygen capacity of modern fish and crustaceans, a relatively low weight specific area in *T. eatoni* may indicate its low oxygen uptake, possibly related to a less active life mode. Exceptionally preserved respiratory structures in the Cambrian deuterostome *Haikouella* are also consistent with a model of countercurrent gaseous exchange, exemplifying the wide adoption of this strategy among early animals.

## 1. Introduction

**Figure 1.**
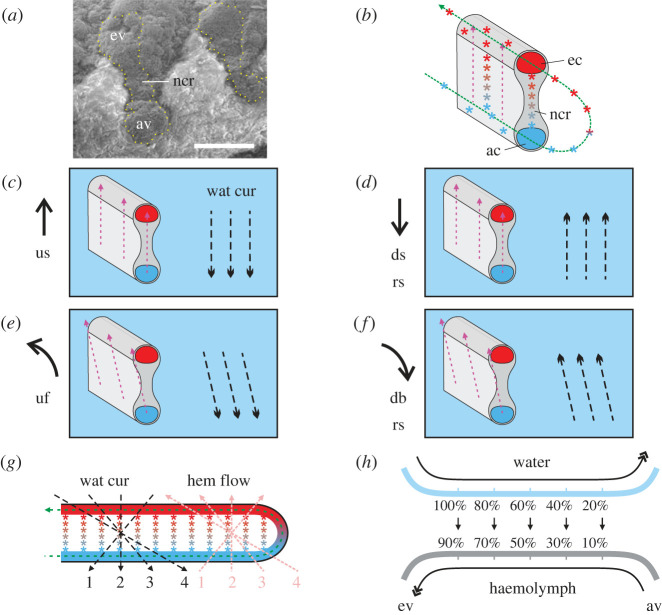
Interaction of haemolymph flow and water current. (*a*) Cross-section of the filament of *T. eatoni*, YPM 204. (*b*) Reconstructed cross-section showing haemolymph flow. (*c*) Upstroke of gill branch creates downward-flowing water current, opposite to upward-directed haemolymph flow. (*d*) Reverse stroke of gill branch creates upward-flowing water current, same direction as upward-directed haemolymph flow. (*e*) Anticlockwise rotation of gill branch creates posteriorly downward-flowing water current, reverse of upward-directed haemolymph flow. (*f*) Reverse stroke of gill branch creates upward-flowing water current, same direction with upward-directed haemolymph flow. (*g*) Any water current flowing downward is always paired with the countercurrent haemolymph flow. (*h*) A countercurrent exchange model shows how the opposite flow exchanges oxygen with gradient difference. Pink dash line with arrow represents the possible routes for haemocyanin from bottom to top side. Black dashed line with arrow represents the water current and its direction. The light blue background represents the water medium. Arabic numbers in (*e*) represent paired countercurrent flow. ac, afferent channel; av, afferent vessel; ec, efferent channel; ev, efferent vessel; db, down backward rotation; ds, downstroke; hem flow, haemolymph flow; ncr, narrow central region; uf, up forward rotation; us, upstroke; wat cur, water current; rs, reverse stroke. Scale bar, 0.05 mm (*a*).

**Figure 2.**
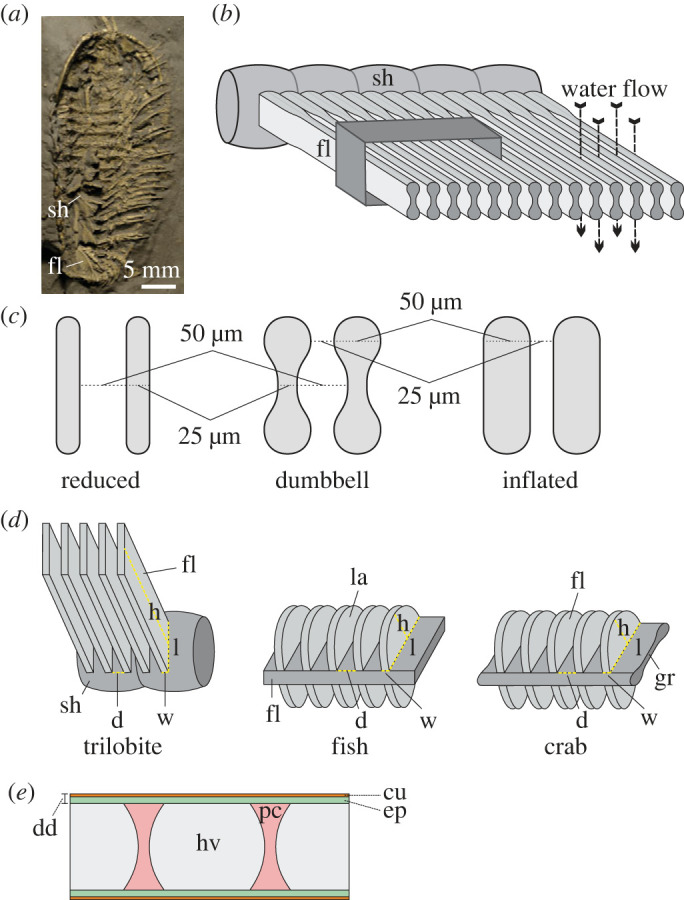
Detailed description of gills. (*a*) Well-preserved gill branches of trilobite *T. eatoni*, GLAHM 163103. (*b*) Reconstructed partial gill branch of *T. eatoni*. The area marked with a black box is the target of computational fluid dynamics (CFD) analysis. Cross-section of the gill filaments shows dumbbell-shaped outline and interspace among filaments. Water currents (marked with black arrows) flow through the interspace between filaments. (*c*) Three types of gill models (10 times actual size) of *T. eatoni* examined in this study: reduced cylinder, dumbbell shape and inflated cylinder. (*d*) Simplified gill models: trilobite, fish and crab showing critical features. (*e*) Simplified cross-section of respiratory filament of crab gills showing the diffusion distance, a possible analogue applicable to trilobite gills. an, anterior; cu, cuticle; d, interfilament or interlamellar distance; dor, dorsal; dd, diffusion distance (or barrier); ep, epithelium; fl, filament; gr, gill raphe; h, height; l, length; hv, haemolymph vessel; in, innerward; la, lamella; ou, outward; pc, pillar cell; sh, shaft; ven, ventral; w, width.

Increasing oxygen availability in Early Palaeozoic ambient seawater has received much attention for its temporal coincidence with the radiation and biodiversification of early metazoans [1–6]. The details of how mechanistically early animals extracted oxygen from seawater are scant, despite this need being critical for the development of highly regionalized bodies and active lifestyles. Respiratory organs, as the primary site of respiratory gas exchange, have been rarely reported in early animals, but the best-preserved specimens can yield insights into their structure and function. Exceptionally preserved Early Palaeozoic arthropods preserve filaments associated with the upper branch whose morphology is consistent with gill function [7–12], but understanding details of how the filaments functioned in oxygen uptake is critical for assessing how organisms responded adaptively to changing physical environments. Recent micro computed tomography (micro-CT) scanning of pyritized specimen of the Ordovician trilobite *Triarthrus eatoni* revealed that filaments on the upper limb branch have a dumbbell-shape consistent with their having a primary respiratory function [7]. This structure is consistent with the gill filaments having had a lower afferent channel and an upper efferent channel (figures 1*a,b*, 2*a,b* and electronic supplementary material, figure S1*a,f–h*,) [11], as recently also confirmed in a well-preserved Silurian trilobite [12]. The distal loop that connected both channels would have enabled continuous flow of haemolymph from the lower vessel to the upper one [13–15] (figure 1*b,g* and electronic supplementary material, figure S1*a–h*), but the system presumably was such that restricted flow within the loop forced haemolymph to flow across the intervening laminae, whose narrow central area was suited to oxygen exchange (figure 1*b,g* and electronic supplementary material, figure S1*h*) via its thin cuticle. Haemocoel in the narrow central region of filaments apparently allowed haemocyanin moving upward to be charged with oxygen and to finally drain into the efferent channel [13–15] (figure 1*b,g* and electronic supplementary material, figure S1*g,h*), the morphology of the filaments being compatible with the inference that the gradient of oxygen concentration gradually changed between the afferent channel and the efferent channel (figure 1*b,g* and electronic supplementary material, figure S1*g,h*).

During strokes of the gill branch, the currents created would have flowed through the space between filaments. The upstroke of the gill branch (efferent channel upper and afferent channel lower) would have created a downward-directed water current (figure 1*c,e*). The first part of the gill to come in contact with incoming water would be the efferent channel, which would be charged with oxygen as a result, followed by the deoxygenated haemolymph in the central laminae and lastly by the afferent channel (figure 1*c,e*). Such a mechanism is well known in modern animals as countercurrent flow (figure 1*h*) and is a highly efficient mode of gill aeration [16,17]. By contrast, the downstroke of the gill branch would have created an upward-directed water current, paralleling the direction of upward flowing haemolymph, yielding less efficient concurrent flow oxygen exchange (figure 1*d,f*). In both cases, regardless of the direction of movement, water was forced into the narrow space between adjacent gill filaments (electronic supplementary material, text).

**Figure 3.**
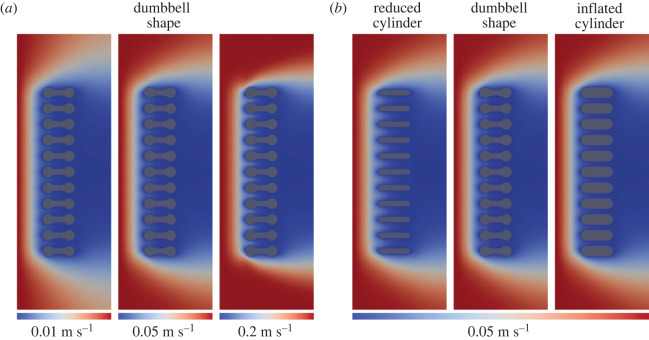
CFD simulations of water current flowing through three gill filament morphologies (shown as cross-sections). (*a*) Three different flow velocities simulated for dumbbell shape model. (*b*) Comparison of three types of models under the same flow velocity. Water flows from left to right. The velocities on the bottom represent the inlet flow velocity. The colour range of the scale bars starts from the 0 m/s to the maximum which is same as the inlet flow velocity.

**Figure 4.**
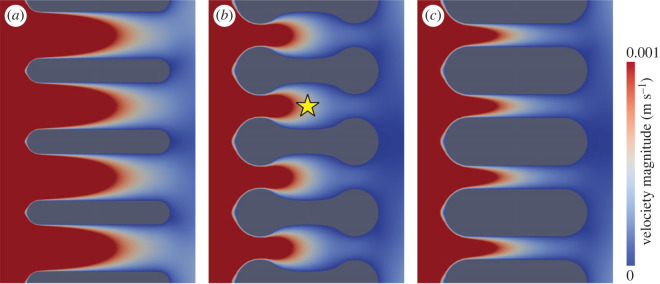
Details of flow velocity among filaments. (*a*) Reduced cylinder model showing uniformly high-speed flows among filaments. (*b*) Dumbbell model showing a buffer zone (marked with a yellow star) centrally that expands water laterally. (*c*) Inflated cylinder model showing uniformly low-speed flow among filaments. Water flows from left to right in this diagram and the velocity of the incoming flow is 0.05 m s^−1^ (inlet flow). The colour range of the velocity map in this figure is, however, visually restricted between 0 and 0.001 m s^−1^, which is designed for displaying the micropatterns of flow paths.

**Figure 5.**
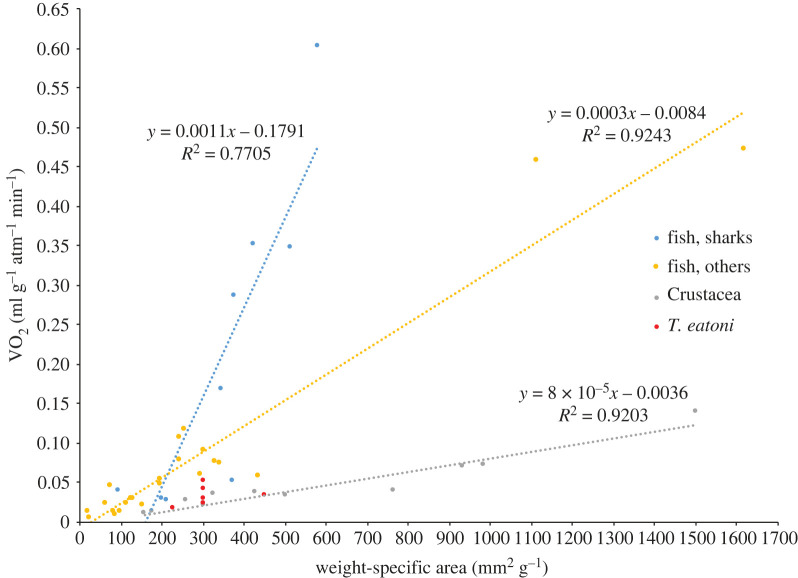
Comparison of oxygen capacity among fish and arthropods. Original data are available in electronic supplementary material, file. VO_2_, oxygen volume.

**Figure 6.**
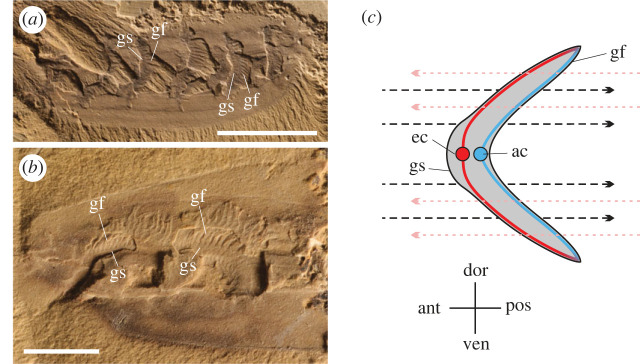
Gaseous exchange in chordate *Haikouella jianshanensis*. (*a*) Well-preserved gills of *H. jianshanesis* bearing a backward curved central supporting structure which is attached with many filaments that have a wide base and pointed end, specimen 146 [24]. (*b*) Gills of *H. jianshanensis*, specimen 088 [24]. (*c*) Reconstruction of gill cross-section of *H. jianshanesis* showing afferent and efferent vessels (based on [24], fig. 2*g*). Black dashed lines with arrows represent possible posterior water current flow. Purple dotted arrow is the oxygen charging path of individual haemocyanin, replacing lower concentration of oxygen with high concentration of oxygen. Suggested water current over the gill filaments from outer surface to inner surface, which permits countercurrent oxygen exchange with haemolymph flowing inside gill filaments. ac, afferent channel; ec, efferent channel; gs, gill supporting structure; gf, gill filament. Scale bars, 5 mm (*a*) and 2 mm (*b*).

In order to test the countercurrent flow model in *T. eatoni*, we use computational fluid dynamics (CFD) [18–21] to simulate water flow around modelled gills (figures 3 and 4). We then estimate the oxygen exchange capacity of *T. eatoni* and compare with that of living fish and decapod crustaceans (figures 2 and 5). Finally, we describe how the gill structure of the distantly related Cambrian deuterostome, *Haikouella jianshanensis*, was also conducive to countercurrent flow, indicating that this respiratory mechanism may have already been widespread early in the evolutionary history of animals (figure 6 and electronic supplementary material, figure S2).

## 2. Material and methods

### 2.1. Materials

Materials described in this paper are housed in the Early Life Institute (ELI), Northwest University, China; The Hunterian Museum, University of Glasgow (GLAHM), UK; Yale Peabody Museum of Natural History (YPM), Yale University, USA. They are available for further research.

The pyritized specimens of *T. eatoni* (figure 2*a*) are from the Beecher's Trilobite Bed of the Upper Ordovician Katian (or Caradocian) Frankfort Shale of upper New York State, USA and the Upper Ordovician Whetstone Gulf Formation (Martin Quarry) [22,23]. The yunnanozoan *Haikouella jianshanensis* (figure 6*a,b*) is from the Chengjiang Biota of the Early Cambrian Heilinpu Formation in Haikou, near Kunming, China [24].

### 2.2. Computational fluid dynamics

#### 2.2.1. Constructing three-dimensional models

The three-dimensional gill models of *T. eatoni* were reconstructed using Blender 2.93.3 (www.blender.org). As the focus of this paper is mainly the interlamellar gaps, we reconstructed a partial gill branch with 11 filaments. Three types of models were reconstructed (figure 2*c*): (i) the dumbbell model, where filaments have swollen ends (corresponding to the channel-hosting end of the inflated marginal bulb of the filament described in Hou *et al*. [11]) to mimic the structure seen in *T. eatoni*; (ii) the reduced cylinder model, where filaments have a rectangular cross-section with the same width as the narrow central region of the dumbbell model; and (iii) the inflated cylinder model, where filaments have a rectangular cross-section with the same width as the swollen end of the dumbbell model. The number of filaments per unit length of the shaft is same in all models, such that the interfilament space is greatest in the reduced model and the smallest in the inflated model (figures 2*c* and 4).

#### 2.2.2. Simulations

The CFD simulations were run with the OpenFOAM v. 2012 (www.openfoam.com). The computational domain (electronic supplementary material, figure S3) is a rectangular box, 1.4 × 0.45 × 0.5 mm (length × width × height), which reaches to an ideal state that does not change the simulated solution even when further enlarging the domain size. The three-dimensional gill models (electronic supplementary material, figure S3) in the domain are 0.15 mm long, 0.8 mm wide and 0.5 mm high. Reynolds number is expressed as

$$Re=\frac{\rho uL}{\mu },$$

where *ρ* is the density of fluid (kg m^3^), *u* is the velocity (m s^−1^), *L* is the characteristic length (m), and *μ* is the dynamic viscosity of fluid (kg m^−1^ s^−1^). The width, 0.8 mm, of the model of 11 filaments including interlamellar space is the characteristic length, serving as the basis for the calculation of Reynolds numbers.

The flow velocity at the inlet is a fixed value and other walls (including outlet) are of zeroGradient condition, conditions that have the least effect on the maximum velocity. The models are set with no-slip boundary conditions. The pressure at inlet and gill models are set with zeroGradient conditions and other walls are set with a fixed value of 100. Three types of flow speed, 0.01 m s^−1^, 0.05 m s^−1^ and 0.2 m s^−1^, are designed based on some studies of simulations [18,21,25–27]. The models and domain were meshed using the snappyHexMesh utility and details of meshing process can be found in Esteve *et al*. [21].

The suggested environmental parameters were selected to reflect *T. eatoni*'s life in a quiet, intermittently dysoxic marine environment in a peripheral foreland basin (electronic supplementary material, table S2). Density and dynamic viscosity of seawater were obtained through the online tool: Pipeng Toolbox (www.pipeng.org). As flow through the gill lamellae in fish is dominantly laminar [28,29] and the small Reynolds numbers in our study (electronic supplementary material, table S2) cannot produce turbulent flow, we selected the pisoFoam solver with the laminar flow model for this study.

### 2.3. Oxygen exchange capacity

Diffusion of gases in crustaceans occurs across thin and uncalcified permeable areas such as the gills in branchial chambers [30] and is directly proportional to gill surface area and inversely proportional to diffusion distance (Fick's Law). Thus, we can use measurements taken directly from the filaments preserved in *T. eatoni* specimens as well as from comparison with living decapod crustaceans to estimate the necessary parameters for calculating and comparing oxygen exchange capacity in *T. eatoni* with modern water-breathing organisms.

#### 2.3.1. Estimates for gill surface area, body mass and cuticle/epithelium thickness of *Triarthrus eatoni*

The cross-section of the filaments is a dumbbell-like shape, with a length of 150 µm and a width of 50 µm at the marginal bulbs (figure 2*d*). Cisne [31] estimated that some gill branches can have up to 100 filaments, but Whittington & Almond [32] estimated the gill branch to bear only approximately 50 filaments. As the filaments close to the proximal and distal ends of the gill branch were relatively poorly preserved, the number of observed filaments is assumed to be far less than its original number. We here consider each gill branch to bear 60 filaments. For a larger-sized *T. eatoni* specimen such as GLAHM 163103 (figure 2*a*), the average height of the filament on the ninth segment is approximately 3.17 mm. Each body segment has paired gill branches, so 120 filaments are counted per segment. The total surface area for the gills of the ninth trunk segment is thus estimated to be approximately 114.12 mm^2^.

As trilobites had clear growth gradients along the body [33], the linear ratios of segment lengths to body length can be obtained. The gill surface area for a given segment was then divided by the ratio of its length to the overall gill-bearing body length, which was estimated as the length from second glabellar furrow to the margin of the pygidium because gill branches are only extended anteriorly to the second glabellar furrow. GLAHM 163103 has a total body length of 3.63 cm, with a gill-bearing body length of approximately 3.0 cm, yielding a total gill surface area of 20.67 cm^2^.

Body volume was reconstructed based on ellipsoidal fit for body shape [34], and *T. eatoni* had a body length twice its exoskeletal width. Here, the ellipsoid was fitted to *T. eatoni* with a length of 3.63 cm and a width of 1.815 cm (figure 2*a*; electronic supplementary material, table S3). Because we do not have direct measurements of the body depth (dorsoventral thickness), we applied three conditions: 1.815 cm (same with the width), 1.21 cm (two-thirds of the width) and 2.42 cm (four-thirds of the width), yielding biovolumes of 6.261, 4.174 and 8.384 cm^3^, respectively. Assuming a reference mass density of 1.1 g cm^−3^, biomass was estimated to be 6.887, 4.592 and 9.183 g, respectively, and used to calculate weight-specific area (see below).

Because the thickness of cuticle and epithelium varies both intraspecifically and among arthropod species, the average thickness of cuticle and epithelium in decapod crustaceans (1.30 and 4.98 µm, respectively, see electronic supplementary material, table S4) was used as estimates of those values in *T. eatoni*. To assess how sensitive the oxygen exchange capacity estimates were to selected thickness values, for a body mass of 6.887 g, we also varied the estimates of thicknesses in the following three ways: (i) cuticle thickness = 0.5 µm, epithelium thickness = 5 µm; (ii) cuticle thickness = 0.3 µm, epithelium thickness = 5 µm; and (iii) cuticle thickness =1 µm, epithelium thickness = 4 µm. The six cases described herein are summarized in electronic supplementary material, table S3.

#### 2.3.2. Estimates of gill surface area, body size and cuticle/epithelium thickness of modern decapod crustaceans and fish

Data on gill surface area, body size and cuticle/epithelium thickness for decapod crustaceans and fish were collected from the literature (electronic supplementary material, table S4). The data on fish consist of two categories: sharks and other fish. For sharks where body mass but not gill surface is reported in the literature, gill surface area was calculated based on the linear regressions from previous studies. Freshwater and marine fish were treated equally, so the difference of oxygen concentrations for both seawater and freshwater was not explicitly considered. However, excluding freshwater organisms did not significantly change the results (see also Discussion).

#### 2.3.3. Krogh's diffusion coefficient

The diffusion coefficient of oxygen varies among different media but shows a constant rate for each [35]. An important parameter is the diffusion barrier (or distance) between ambient water and internal blood or haemolymph. In vertebrates, the diffusion barrier is epithelium only, but in invertebrates the gill filaments have an integrated diffusion barrier consisting of an external cuticle layer and an internal epithelium layer. For the epithelium layer, we selected the Krogh's diffusion coefficient of muscle, as used in comparable studies [36], 0.14 ml O_2_ *µ* cm^−2^ atm^−1^ min^−1^, as the basis for calculation, where the *µ* is the diffusion distance. As invertebrates have an integrated diffusion distance across both cuticle and epithelium, we needed to combine both different types of diffusion coefficient in the calculation. Chitin has a relatively low diffusion coefficient, 0.013 ml O_2_ *µ* cm^−2^ atm^−1^ min^−1^, for the cuticle diffusion. We used the following formula to calculate the diffusion coefficient:

$${\mathrm{DO}}_{2}=\frac{1}{0.14^{-1}*(E/(C+E))+0.013^{-1}*(C/(C+E))},$$

where DO_2_ is the diffusion coefficient, *E* the thickness of the epithelium layer, *C* the thickness of the cuticle layer. This formula is modified from Aldridge & Cameron [37] to limit the diffusion path to only epithelium and cuticle layers.

The diffusion distance of oxygen to blood or haemolymph across the barrier may be complicated in some water breathing organisms by internal supporting structures such as pillar cells [36] which obstruct the passage of oxygen, extending the distance of its journey into the body. However, because there is no evidence of pillar cells in *T. eatoni*, we simply used the thickness of epithelium or the integrated thickness of cuticle and epithelium as the diffusion distance, and did not consider the effects of such cells.

#### 2.3.4. Oxygen capacity

The original formula for the oxygen capacity is VO_2_ = DO_2_ × cm^2^/*µ* [35,36]. Here we replaced the surface area, cm^2^, with wet weight-specific area, mm^2^ g^−1^, where the g is the mass of the body to accommodate the varied data published for different animals. The new formula is VO_2_ *=* DO_2_ × 0.01 mm^2^ g^−1^*/µ*, and the final result is the oxygen volume in ml O_2_ g^−1^ atm^−1^ min^−1^. The final data for *T. eatoni* as well as modern fish and decapod crustaceans is summarized in electronic supplementary material, table S4.

#### 2.3.5. Resistance of interfilament channel

Water flow speed is an important factor for the oxygen uptake in aquatic animals. Structure design and its effect must be coordinated well and then can efficiently serve for the animals. Resistance is thus a key to reveal the mechanisms behind the structures. The Hagen–Poiseuille equation below describes the resistance of the interlamellar (for fish) or interfilament (for arthropod) channel [38].

$$R=\frac{12ul}{d^{3}h},$$

where *u* is the dynamic viscosity of the water, *l* is the length of interlamellar (or interfilament) channel, *d* is the diameter of the channel, *h* is the height of the lamella or filament (figure 2*d*). The short length or the long diameter of the interfilament channel will produce less resistance to the water flow, whereas the long length and the short diameter of the interfilament channel will increase water flow resistance.

## 3. Results

### 3.1. Computational fluid dynamics simulation of gill function

The reduced cylinder model, the dumbbell model and the inflated cylinder model (figure 2*c*) all show that the velocity of water moving between filaments slows as it approaches the filaments and forms a residual drag pattern after passing through the filaments (figures 3 and 4). The water speed is faster near the margins of the model. The size of the interlamellar gap is positively related to the speed of flow between the filaments, being high in the reduced cylinder model and low in the dumbbell and inflated cylinder models (figures 3 and 4 and electronic supplementary material, figure S4). However, in all models, in the interlamellar gap, the water speed decreases from one end of the model, where oxygen-charged water is encountered, to the opposite end of the model. The three gill models display contrasting flow patterns (figures 3 and 4), but flow velocity always increases toward the centre of the interfilament space, away from frictional slowing associated with the walls of the gills. The reduced cylinder model has a uniformly high flow velocity among filaments (figure 4*a*) and the inflated cylinder model has uniformly low velocity among filaments (figure 4*c*). By contrast, the dumbbell model results in a flow velocity similar to that of inflated cylinder model (figure 4*b*). The swollen ends of the gill filaments reduced the adjacent flow speed, especially in the narrow central region of the dumbbell. In this portion, where the distance between adjacent filament membranes is greatest, the speed of the flow decreased. The constrictions at each end of the dumbbell thus impede the passage of water within the central elliptical cavity, pressing it against the membranes and facilitating oxygen exchange within this region. Compared with the reduced cylinder model, the swollen ends of the dumbbell slow the flow, but in contrast to the inflated cylinder model, the elliptical shape of the central zone in the dumbbell model increases the volume between adjacent gill filaments in this area. The velocity of the water continues to decrease as it is expelled through the narrow gap between the opposite swollen end of the dumbbell.

### 3.2. Oxygen exchange capacity and flow resistance in *Triarthrus eatoni*

The six modelled scenarios of *T. eatoni* gill structure (electronic supplementary material, table S3) show little variation in total oxygen capacity, and all position *T. eatoni* among the values shown among modern decapod crustaceans (figure 5 and electronic supplementary material, figure S5, table S4 and text). The volume of oxygen is negatively correlated to the thickness of cuticle, descending from case 3 (0.3 µm cuticle), case 2 (0.5 µm cuticle), case 4 (1 µm cuticle) and the rest (1.3 µm cuticle) (electronic supplementary material, table S4). However, the weight specific area (gill surface area per gram: mm^2^ g^−1^) (300–450 mm^2^ g^−1^) of *T. eatoni* (figure 5) is far less than those of aquatic decapods with the weight specific area ranging from 500 to 1400 mm^2^ g^−1^ but similar to those above tide with the weight specific area ranging from 280 to 640 mm^2^ g^−1^ [39].

In *T. eatoni*, the average height of the filaments (*h*) is approximately 10 times the filament length (*l*), resulting in a large ratio of height to length. For a fixed value of channel diameter (*d*), the distinctly high height of filaments produces less resistance to the water flows when compared with the low height, or long length, of filaments, which would result in a small ratio of height to length.

## 4. Discussion

### 4.1. Implications of the computational fluid dynamics simulation

All CFD models together suggest that flow velocity decreased markedly between adjacent filaments (figures 3 and 4). The dumbbell-shaped filaments created a ‘buffer zone’ (figure 4*b*) in which the water volume is increased compared with the inflated cylinder model under almost the same category of velocity even as the water flow velocity continued to decrease through the gap between the swollen ends of the adjacent dumbbell-shaped filaments. The swollen ends have been suggested to prevent trilobite gill filaments from collapsing, as well as housing the swollen afferent and efferent vessels [11,13,14,40], but our analysis suggests that they also functioned to reduce water flow speed in the vicinity of the respiratory surfaces. As impeding the velocity of water flow still needs a high strength, the support function with high strength is probably the dominant role for the swollen ends. Contrary to the cylinder-shaped outline, the dumbbell shape with a curved surface is clear evidence of increasing gill surface area. Accordingly, the dumbbell-shaped filaments mechanically improved respiration efficiency with only modest deviation from a simple, cylindrical filament shape, just as in fish gills where interlamellar distance has evolved to an optimal state for maximizing oxygen transfer [26].

### 4.2. Optimal solution between gill surface area and flow resistance

Fish that are active swimmers show increased filament length and a large number of secondary folds (or lamellae) compared with those that are sluggish [41]. Increased lamellar length or lamellar packing (i.e. decreasing interlamellar space) amplifies resistance to the flowing water, while increasing the lamellar height or the filament length decreases resistance [38,41]. Contrary to the condition in fish, the elongate filaments of trilobites represent the main site for oxygen uptake (figure 2*d*). The gills of *T. eatoni* have a distinctly long shaft that in this trilobite species extends far beyond the exoskeletal margin (figure 2*a* and electronic supplementary material, figure S1*a*). In crab gills, the filaments are mostly semicircular shape, with their height less than their length [11,13] (figure 2*d*), and in most fish and crabs the gills are sealed in the branchial chambers, of which the latter limits the length of the filaments [38]. In trilobites, the gills are open to the ambient water, but when imbricated (gill branches inclined but stacked nearly vertical with respect to each other) they had limited space for lateral water flow between adjacent gill branches [11], and filaments with greater length would have provided more resistance. Maintaining gill area without increasing the area of overlap may have been achieved by increasing the height of the gills, which decreased the resistance of water flowing through the filaments because of the decreasing ratio of filament length to height (figure 2*d*). The replacement of the open-type gill filament in trilobites by the closed-type gill in crustaceans suggests increased functional specialization in the more derived group. The trade-off between gill surface area and resistance in *T. eatoni* seems to have been optimized. Expansion of the respiratory area beyond the protection of the exoskeleton in *T. eatoni* may suggest that the need to maximize respiratory surface area [42] was paramount to those of protection against predators. This is consistent with this species occupying a low predator pressure environment at the margins of trilobite respiratory habitability (electronic supplementary material, text).

The countercurrent gaseous exchange mechanism is capable of absorbing up to 90% of dissolved oxygen [43] in fish and crustaceans. Nonetheless, the efficiency of the countercurrent exchange mechanism varies among organisms depending on differences in water pressure gradient, conductivity and flow speed; tissue perfusion; delivery of haemocyl-bound oxygen to the tissues; and the oxygen loading capacity of the haemolymph or blood [44–46]. The activity of these animals is positively correlated with their oxygen diffusion capacity [47]. In crustaceans, the limited oxygen absorption capacity suggests a less active life mode, when compared with fish (figure 5). Lower efficiency of countercurrent gaseous exchange in modern crabs compared with that of modern fish is mainly due to the properties of the diffusion barrier (e.g. its cuticular thickness and density) and not to a less effective countercurrent flow system *per se* [48]. The oxygen capacity modelled in *T. eatoni* is consistent with the negative correlation between the total volume of oxygen extracted and the thickness of the diffusion barrier (discussed in §3.2). If a less permissive barrier characterized all arthropods (including trilobite data discussed in §3.2), this may have been offset in trilobites by increasing the number and size of the gill branch and/or respiratory filaments [49]. With respect to the modern aquatic decapods, *T. eatoni* also has a relatively low weight specific area (figure 5) and is particularly near the lower values of oxygen capacities among marine decapods (electronic supplementary material, figure S5). This may indicate that oxygen uptake in *T. eatoni* was relatively low, possibly related to a less active life mode (electronic supplementary material, text) and a slow growth history [50].

### 4.3. The presence of countercurrent gaseous exchange in other Palaeozoic animals

In the trilobite *T. eatoni*, ‘countercurrent flow' was operative during upward movement of the limb (figure 1*c,e*), while during downward movement concurrent flow exchange applied (figure 1*d,f*). The countercurrent gaseous exchange mechanism was also evidently applied in the gill system of the deuterostome yunnanozoan *Haikouella jianshanensis* of the Chengjiang Biota (figure 6). *Haikouella's* gills bear a central arch-like supporting structure, curved posteriorly and connected to the ventral blood vessels [24]. Closely arranged paired filaments are attached on the lateral and possibly posterior surfaces of the supporting structure. The triangular filaments are flat and taper distally from its wide base. Paired filaments appear perpendicular to the supporting structure (figure 6*b*). The reconstructed cross-section of the *Haikouella* gill shows the possible haemolymph circulation. The flat filaments leave the vessels near the lateral edges of the gill-supporting structure. Afferent channels are interpreted to be those on the inner side of the paired gill filaments and the efferent channels are inferred to be located at the outer side (figure 6*c*), just as in modern fish gills [48]. In the stem deuterostome vetulicolians, unidirectional water currents have been suggested to flow in through the mouth and out via the gill slits [51]. We suggest that *Haikouella* may have employed a similar unidirectional flow and also employed the countercurrent exchange mechanism. The closely comparable morphology of the gill system in modern fish and in yunnanozoans indicates operation of the countercurrent exchange mechanism in Early Cambrian deuterostomes (electronic supplementary material, figure S2, table S1 and text).

The biovolume (or body size) of animals is limited by the circulatory system and respiratory medium [52] and these factors apparently assumed particular importance in early metazoan history, during which ambient levels of oxygen were lower than later in the Palaeozoic and thereafter [53,54]. The ability to achieve oxygen concentration in the efferent vessel almost as high as the ambient oxygen level may have allowed yunnanozoans and trilobites to occupy a broad range of habitats and possibly to evolve multiple feeding modes, which are considered to play a key role in the development of early metazoans [52].

## Data Availability

All the specimens described in this paper are in the collections of the Early Life Institute (ELI), Northwest University, China; Hunterian Museum, University of Glasgow (GLAHM); and the Yale Peabody Museum of Natural History (YPM) of Yale University, and are available for further research. The three-dimensional models (.stl files) and the essential files for running CFD through OpenFOAM are available at the AMNH Digital Repository: https://doi.org/10.5531/sd.paleo.11 [55]. The data are provided in electronic supplementary material [56].
